# A Survey of Bluetongue Infection and Associated Risk Factors among the One-Humped Camel (*Camelus dromedaries*) in Gadarif State, Eastern Sudan

**DOI:** 10.1155/2021/6613217

**Published:** 2021-03-24

**Authors:** Hatim H. Abraheem, Amira M. Elhassan, Mohammed O. Hussien, Khalid A. Enan, Azza B. Musa, Abdel Rahim M. El Hussein

**Affiliations:** ^1^Central Veterinary Research Laboratory (CVRL), Animal Resources Research Corporation (ARRC), P.O. Box 8067, El Amarat, Khartoum, Sudan; ^2^Central Laboratory, Ministry of Higher Education and Scientific Research, P.O. Box 7099, Khartoum, Sudan

## Abstract

Bluetongue (BT) is an infectious, noncontagious, vector-borne viral disease that affects wild and domestic ruminants transmitted by *Culicoides* spp. A cross-sectional study was carried out during the period 2016-2017 in Gadarif state. A total of 276 sera samples were collected from camels in six localities of Gadarif state, eastern Sudan, to investigate bluetongue virus (BTV) seroprevalence and associated risk factors of BTV infection including age, sex, breed, locality, and ecology of the region. Enzyme-linked immunosorbent assay (ELISA) was used for estimation of BTV seroprevalence rate. The overall BTV seroprevalence rate was 96.7% in the study area ranging from 93.5% to 100% in six screened localities with no significant differences. The findings revealed similar BTV seroprevalence rates in both males and females, but high rates were found in age group of less than one year and two to three years with estimated 100%. However, the lowest seroprevalence was found in the age group of five to four years with estimated BTV to be 92.3%. BTV seropositivity was not found to be statistically associated with examined different camel breeds which revealed 93%, 94.4%, 97.6%, and 97.8% seroprevalence in Bushari, Rashide, Arabi, and Anafi, breeds, respectively. Epidemiology of BTV assessment according to the ecology of the area showed high BTV seroprevalence in desert and savanna with estimated 100% and lower BTV seroprevalence in arid and rich savanna with estimated 94.8% and 95.7%, respectively. There was no significant association between BTV ELISA positivity and sex, breed, and ecology of the area.

## 1. Introduction

The total camel population in the world is 19 million, of which, 17 million are dromedaries and 2 million are Bactrian [1]. The population of camels in Sudan is 3.3 million [2, 3] of which 564,756 heads are in Gadarif state. Many tribes in different parts of the Sudan depend entirely on camels for their livelihood [4, 5]. Several studies in various countries have reported on the widespread infection of camels with bluetongue virus. These infections are mostly unapparent infection with no noticeable signs or symptoms. Bluetongue virus (BTV) is a double-stranded RNA virus (family *Reoviridae*, genus *Orbivirus)* that causes bluetongue in ruminants with at least 28 recognized serotypes. Clinical signs of the disease include fever, nasal discharge, excessive salivation, facial edema, ulceration, cyanosis of tongue (bluetongue), coronitis, and skeletal muscle damage. Severe clinical signs are observed in sheep, and mild symptoms are usually shown in cattle, goat, camelids, and carnivores [6–8]. *Culicoides* midges are the main vectors of the virus, with *C. imicola* being the main vector species in Africa and southern Europe.

Earlier, Abu [9] conducted a serological survey in the one-humped camel in Sudan using agar gel immune-diffusion (AGID) test which revealed 40%, 19.2%, 8.7%, 4.3%,6%, and 0% positivity in Al Fashir, Nyala, Tampol, Kassala, Gadarif, and Sennar, respectively, with an overall seroprevalence of 16.6%. On the other hand, seroprevalence surveys of BTV in camel conducted in Khartoum and Kassala states revealed 66.8% and 12.7% seroprevalence rates, respectively [10, 11]. However, other serological surveys showed that the antibodies against BTV were widespread in livestock species such as goats, cattle, and sheep in the country [9, 11, 12].

The aim of this investigation was to determine the prevalence of BTV antibodies among different camel breeds and assess the risk factors predisposing the animals to BTV infection in Gadarif state, eastern Sudan.

## 2. Materials and Methods

### 2.1. Study Area

**Figure 1:**
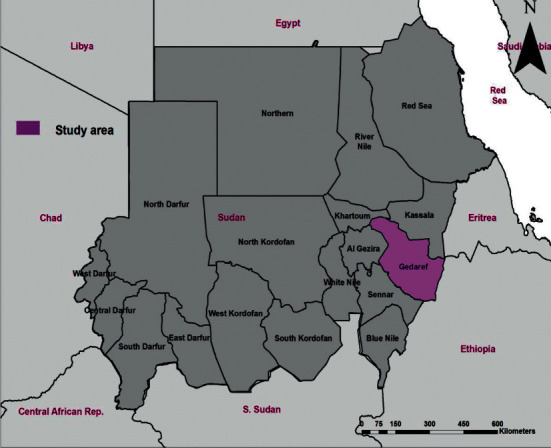
Map of Sudan showing the study area (Gadarif state) where serum samples were collected.

This study was carried out in Gadarif state which lies between 16.14° altitude and 33.35° longitude (Figure 1). The climate is hot in the summer. The rainy season extends four months, with an average of annual rainfall of 700 to 900 mm. Average temperature ranges between 21.2 and 36.5°C, whereas average relative humidity is around 43%. Gadarif state is boarded by five states and one neighboring country. The study area is divided into six localities.

### 2.2. Study Design

This is a cross-sectional study that was carried out during the period 2016-2017. A total of 276 samples were randomly collected from camels in six localities including three localities from north section (Gadarif, central Gadarif, and Butana) and one from east (Fashaga). The method was random selection of villages from each of the 6 localities mentioned above, and the strategy depends on the covering of all localities. Finally, simple random sampling was applied to choose the camels from each village. All camels included in this study were aged <1 year to >5 years. Camels sampled were of both sexes and from local breeds including Bushari, Anafi, Arabi, and Rashide rearing in different ecological areas including desert, arid, savanna, and rich savanna.

### 2.3. Collection of Blood Samples

Blood samples were collected from jugular vein using plain vacutainer tubes with a needle holder after cleaning the puncture area with 70% alcohol. A volume of 5 to 7 ml of blood was collected aseptically from each animal, and tubes were left to stand overnight in a refrigerator for serum separation. Serum was decanted in capped vials and frozen at −20^o^C until transported to the Central Veterinary Research Laboratory for screening.

### 2.4. Risk Factors

Risk factors investigated in this study included age, sex, breed, locality, and ecology of the area (which were recorded with each serum sample) and subsequently analyzed using Statistical Package for the Social Sciences (SPSS). Based on ecological features, the study area was divided into four categories, namely, desert, arid, savanna, and rich savanna.

### 2.5. Laboratory Analysis for Samples

Indirect enzyme-linked immunosorbent assay (iELISA) was performed using commercially available BTV kit for the detection of specific IgG antibody (Ingizme, France) according to the manufacturer's instructions. In brief, ELISA was performed in 98-well antigen-coated microplate. The incubation was performed for 30 min at room temperature, the plates were then washed six times, conjugate was added and incubated for 45 min, and then the substrate was added and incubated for 10 min. The reaction was stopped using stop solution, and then results were read using ELISA reader (Biochrome, England) set at 630 nm.

### 2.6. Statistical Analysis

Statistical Package for the Social Sciences (SPSS) software (version 16.0) was used for the analysis of the results. Association between the outcome variables and its risk factor was firstly screened in univariate analysis. Then, multivariable model for the outcome variable was constructed, and BTV infection was considered as dependent variable and the risk factors as independent variables. Finally, odd ratios 95% at confidence interval were calculated. *p* ≤ 0.05 p indicated significant association.

## 3. Results

The overall BTV seroprevalence rate in camels was 96.7%, ranging from 93.5% to 100% in the six screened localities. No significant association (*p* > 0.05) between BTV seropositivity and age, sex, breed, and locality was found. In the surveyed camel, females and males had similar seroprevalence with estimated 96.7%, unlike age groups which had different BTV seroprevalence rates ranging from 92% to 100%. In the current study, the most infected camel breeds were Anafi (97.8%) and Arabi (97.6%) compared with Bushari (93.0%) and Rashide (94.4%) (Table 1). BTV seropositivity assessment according to the ecological area showed high BTV antibody prevalence in desert and savanna with estimated 100% and lower BTV prevalence in arid and rich savanna with estimated 94.8% and 95.7% seroprevalence, respectively (Table 1).

## 4. Discussion

In the present study, no clinical signs were observed in camels, although the overall BTV seroprevalence rate in camels in Gadarif was 96.7% (ranging from 93.5% to 100% in different localities) which was higher than that reported (78.6%) by Elmahi [13] in the neighboring Kassala state and (66.8%) by Saeed and Aradaib [10] in Khartoum state. However, earlier seroprevalence surveys showed much lower prevalence in Kassala State (12.7%) by Hassanin [11] and 6% by Abu [9] in Gadarif state. Both of these authors used the much less sensitive agar gel immuno-diffusion test (AGID). On the other hand, Chandel et al. [14] in India found the estimated seroprevalence rate to be 6.9% in healthy camels and 12.6% in sick camels. These findings could be tentative evidence of existence of clinical signs of BTV infection in camels, but more work is needed in Sudan to prove it. Our risk assessment studies indicated that there was no significant association between BTV seropositivity in camels and sex, age, breed, locality, and ecology of the area.

The overall seroprevalence herein reported was higher compared with the seroprevalence rate in Saudi Arabia, Algeria, and Iran which was 25.7%, 21%, and 67.8%, respectively [15–17]. This could be due to the degree of exposure of camels to the vectors as well as management practice of keeping them for a long time. The lower seroprevalence of BTV (66.6%) in camels in Khartoum detected by Saeed and Aradaib [10] compared to our study could be due to the rapid intake of camel in Khartoum state by slaughtering or export as well as low level of vector and/or BTV activity.

There was no significant association (*p* > 0.05) between BTV infection rate noted in this study and ecology of the area. This is in concordance with the findings reported by Elmahi [13] in camels in Kassala state, eastern Sudan. However, the high seroprevalence of BTV in camels in the desert and arid areas in our study (100%) is unexpectedly very high as these areas may not be suitable to support large populations of *Culicoides* midges. This could be explained by existence of very localized foci, such as around seasonal water bonds, of *Culicoides* breeding and survival, or due to movement in and out of the areas. Otherwise, other ways of acquiring the infection such as contact and transplacental transmission as occuring in cattle and goats [18, 19] may be working for camels in these areas especially around seasonal water bonds where large numbers of animals closely congregate. Such variables should deserve further investigation.

Our current study revealed no significant association (*p* > 0.05) between the BTV seroprevalence rate and age of animals. This is in line with the finding reported by Elmahi [13] in camels in Kassala state.

## 5. Conclusions

It could be concluded that BTV antibodies are highly prevalent in camels in Gadarif state. No significant association was detected between BTV seropositivity and the risk factors predisposing the animals to the disease. It is recommended that entomological surveillance of biting *Culicoides* midges involved in the transmission of BTV, and studies of their ecology and epidemiology in the area should also be carried out to better forecast and respond to BT disease in Gadarif state, Sudan.

## Figures and Tables

**Table 1:** Multivariate analysis for the association between potential risk factors and BTV seropositivity among camels in Gadarif state, eastern Sudan, using chi-square test.

Factor	Animals tested	Animals affected (%)	*p* value	Odds ratio	95% CI
					Lower-upper
Locality					
Gadarif	46	43 (93.5)	Ref	—	—
Central Gadarif	19	18 (94.7)	0.848	1.256	0.122–12.896
Gor Aisha	46	46 (100)	0.998	—	—
Fashaga	32	31 (96.9)	0.513	2.163	0.215–21.785
Butana	41	41 (100)	0.998	—	—
Al-Rahad	92	88 (95.7)	0.586	1.535	0.329–7.165

Sex					
Female	213	206 (96.7)	Ref	—	—
Male	63	61 (96.8)	0.965	1.036	0.210–5.119

Age (years)					
0-1	19	19 (100)	0.998	—	—
1-2	33	32 (97.0)	0.406	2.667	0.264–26.938
2-3	17	17 (100)	0.998	—	—
3-4	21	20 (95.2)	0.667	1.667	0.162–17.100
4-5	39	36 (92.3)	Ref	—	—
>5	147	143 (97.3)	0.165	2.979	0.638–13.909

Breed					
Bushari	43	40 (93.0)	Ref	—	—
Arabi	124	121 (97.6)	0.186	3.025	0.587–15.590
Anafi	91	89 (97.8)	0.196	3.337	0.537–20.757
Rashide	18	17 (94.4)	0.838	1.275	0.124–13.147

Ecology					
Desert	41	41 (100)	0.998	—	—
Arid	97	92 (94.8)	Ref	—	—
Savanna	46	46 (100)	0.998	—	—
Rich savanna	92	88 (95.7)	0.795	1.196	0.311–4.598

## Data Availability

The data used to support the findings of this study are available from the corresponding author upon request.
